# An *in vitro* Model of Human Retinal Detachment Reveals Successive Death Pathway Activations

**DOI:** 10.3389/fnins.2020.571293

**Published:** 2020-11-26

**Authors:** Jelena Potic, Martial Mbefo, Adeline Berger, Michael Nicolas, Dana Wanner, Corinne Kostic, Alexandre Matet, Francine Behar-Cohen, Alexandre Moulin, Yvan Arsenijevic

**Affiliations:** ^1^Department of Ophthalmology, University of Lausanne, Jules-Gonin Eye Hospital, Fondation Asile des Aveugles, Lausanne, Switzerland; ^2^Clinic for Eye Diseases, Clinical Center of Serbia, Belgrade, Serbia; ^3^Department of Ophthalmology, Faculty of Medicine, University of Belgrade, Belgrade, Serbia; ^4^Department of Ophthalmology, Institut Curie, Université de Paris, Paris, France; ^5^INSERM U 1138, Centre de Recherches des Cordeliers, Université Paris Descartes, Université Pierre et Marie Curie, Paris, France

**Keywords:** human retina, *in vitro* model, retinal detachment, photoreceptor death, cell death pathways

## Abstract

**Purpose:**

was to create an *in vitro* model of human retinal detachment (RD) to study the mechanisms of photoreceptor death.

**Methods:**

Human retinas were obtained through eye globe donations for research purposes and cultivated as explants. Cell death was investigated in retinas with (control) and without retinal pigment epithelium (RPE) cells to mimic RD. Tissues were studied at different time points and immunohistological analyses for TUNEL, Cleaved caspase3, AIF, CDK4 and the epigenetic mark H3K27me3 were performed. Human and monkey eye globes with retinal detachment served as controls.

**Results:**

The number of TUNEL-positive cells, compared between 1 and 7 days, increased with time in both retinas with RPE (from 1.2 ± 0.46 to 8 ± 0.89, *n* = 4) and without RPE (from 2.6 ± 0.73 to 16.3 ± 1.27, *p* < 0.014). In the group without RPE, cell death peaked at day 3 (*p* = 0.014) and was high until day 7. Almost no Cleaved-Caspase3 signal was observed, whereas a transient augmentation at day 3 of AIF-positive cells was observed to be about 10-fold in comparison to the control group (*n* = 2). Few CDK4-positive cells were found in both groups, but significantly more in the RD group at day 7 (1.8 ± 0.24 vs. 4.7 ± 0.58, *p* = 0.014). The H3K27me3 mark increased by 7-fold after 5 days in the RD group (*p* = 0.014) and slightly decreased at day 7 and was also observed to be markedly increased in human and monkey detached retina samples.

**Conclusion:**

AIF expression coincides with the first peak of cell death, whereas the H3K27me3 mark increases during the cell death plateau, suggesting that photoreceptor death is induced by different successive pathways after RD. This *in vitro* model should permit the identification of neuroprotective drugs with clinical relevance.

## Introduction

Retinal detachment (RD) is one of the most common causes of photoreceptor cell death worldwide (Subramanian and Topping, 2004). Photoreceptor cells die when they are physically separated from the underlying retinal pigment epithelium (RPE) and choroidal vessels, which provide metabolic support to the outer layers of the retina. Although various pathological changes occur in detached retinas (Anderson et al., 1981; Lewis et al., 1994; Jablonski et al., 2000), studies on experimental models have shown that photoreceptor cell death is immediately induced, as early as 12 h after the RD event, and peaks after 2 or 3 days (Cook et al., 1995; Hisatomi et al., 2001; Arroyo et al., 2005). This indicates that an early intervention could potentially preserve the photoreceptors, but delayed care may lead to photoreceptor loss and visual acuity deterioration as consequences. Photoreceptor degeneration processes are currently not fully understood. In rodent models of RD, apoptotic and cell death factors such as apoptosis inducing factor (AIF) and RIPK3 were detected 3 days post detachment (Hisatomi et al., 2001, 2002; Trichonas et al., 2010). The contribution of the apoptotic pathway to photoreceptor death was clearly demonstrated in rodent models of RD by the anti-apoptotic intraperitoneal injection treatment of Bcl-X-TAT fusion protein, which markedly decreased photoreceptor death, 3 days post detachment (Hisatomi et al., 2008). AIF release and Caspase-9 activation were observed in RD human retinas (Hisatomi et al., 2008), but the use of anti-apoptotic factors such as Z-VAD in rodent models of RD did not significantly reduced photoreceptor death (Murakami et al., 2013), suggesting that other pathways are also activated during the cell death process.

Photoreceptor cell death also occurs during the disease course of other retinal disorders such as retinitis pigmentosa (RP), age-related macular degeneration (AMD), and after light-damage of the retina (Wenzel et al., 2005; Ambati and Fowler, 2012; Sahaboglu et al., 2013). Although the causes and clinical characteristics of each retinal disorder differ, accumulating evidence suggests that some molecular pathways leading to photoreceptor cell death appear to be shared by these diseases at least in part (Sancho-Pelluz et al., 2008; Murakami et al., 2013; Zencak et al., 2013). As example, cell cycle proteins play a key role in neurons committed to cell death in several neurodegenerative diseases (Herrup and Yang, 2007) including inherited retinal dystrophies as well as acute retinal injury by light (Al-Ubaidi et al., 1992; Vincent et al., 1996; Yang et al., 2001; Nguyen et al., 2003; Höglinger et al., 2007; Zencak et al., 2013), where post-mitotic neurons start to express nuclear cyclin-dependent kinase 4 (CDK4), usually implicated in the re-entry into the cell cycle. The *in vitro* CDK4 inhibition in retina explants of a mouse model of RP induced by roscovitine evidenced the role of this kinase in the cell death process (Zencak et al., 2013). Loss-of-function studies revealed that downstream and upstream molecular actors, E2F1 and BMI1, respectively, contribute also to cell death induction, BMI1 having the most important role.

Interestingly, and in accordance with the potential action of BMI1, epigenetic modifications recently appeared to play a role during retinal degeneration – such as DNA methylation (Wahlin et al., 2013; Farinelli et al., 2014) and histone modifications (Corso-Díaz et al., 2018; Zheng et al., 2018). An interference with DNA methylation using a DNMT (DNA methyltransferases) inhibitor *in vitro* in retinal explants showed a neuroprotective action on photoreceptor survival during the degenerating process (Farinelli et al., 2014). The authors also demonstrated an increase in DNA methylation in dying photoreceptors in several RP animal models analyzed, suggesting DNA hypermethylation as a common denominator in the photoreceptor degeneration pathway. Their findings thus propose a complex relation between DNA methylation and retinal degeneration, which may include both protective actions and disease-driving elements (Farinelli et al., 2014). A similar observation was postulated with the appearance of the H3K27me3 mark, which presents a small increase during the degenerative process in the *Rd1* retina, a mouse model of retinitis pigmentosa (Zheng et al., 2018), but the cell type expressing this mark was not determined.

In this study, we investigated human retinas *in vitro*, in order to have a better comprehension of the cell death mechanisms that may occur in patients affected by RD. Our work aims to reveal whether apoptosis, cell cycle reentry and epigenetic modifications are also involved in an *in vitro* model of human RD.

## Materials and Methods

### Biological Samples

Retina tissues from human eye globes were obtained following procedures conformed to the tenets of the Declaration of Helsinki for biomedical research involving human subjects and according to the ethical approval (protocol No 340-15) and Swiss law. Human globes received from the Lausanne Eye Bank had been assessed as unsuitable for cornea transplantation. The overall health of the donor before death was considered and tissue from donors with previous history or treatment that might damage the retina was rejected.

We also used sections of *Macaca fascicularis* eye globes obtained for a previous experiment we performed for a gene therapy study, including controls injected with vehicle (TSSM) in which a retinal detachment was observed (for the animal origin and experimentation authorization, please refer to Matet et al., 2017).

### Protocol for Preparing Human Retinal Explants

Following the donor cornea retrieval, the posterior segment was recovered; the vitreous was removed, then the sclera and retina were cut in a flower shape with four cuts. Next the retina was detached from the RPE, choroid and sclera, in order to obtain the *in vitro* model of RD. For the control samples, to maintain the RPE, Proteinase-K incubation during 20 min was performed as previously described in Zencak et al., 2013. The peripheral retina was dissected into small pieces of a minimum of about 5 mm × 5 mm. Retinal explants were placed on Millipore membrane inserts (Millicell Cell Culture Plate Inserts, Merck Millipore, Merck, Darmstadt, Germany), which were then placed in a 6 well plate (Nalgene Nunc, Rochester, NY, United States), with 1.5 mL of medium in each well. The R16 medium was first used, but we observed little cell death in this condition. The medium used contained: 1% B27, 1% FBS, 1% P/S, 97% DMEM/F12. The explants were maintained at 37°C, 5% CO2, and the medium was changed every 48 h. During this protocol, fixation was performed 1, 3, 5 and 7 days after dissection using 4% paraformaldehyde for 120 min. The explants were cryopreserved in 30% sucrose, then mounted using Yazulla [water solution containing 22.5% of egg albumin (Applichem) and 2.3% gelatin (Merck)] and subsequently frozen at −20°C.

### Retinal Explant Cultures

In order to mimic retinal detachment, the retinal explant of each donor sample was held in the medium without RPE, and with RPE for the control group [as previously described for mouse retina explants (Zencak et al., 2013)]. The presence of RPE was ascertained by a regular pigmented epithelium with normal nuclei. No choroid or sclera was present in the samples analyzed in this work. Indeed, we observed that the presence of the choroid provoked marked cell death (data not shown).

Four retinal explants fulfilled the inclusion criteria (*n* = 4). All 4 explants were negative for known retinal diseases. Each biological sample was divided in two groups: RD group (without RPE) *n* = 4, and control group (with RPE) *n* = 4, and served for all the time points analyzed.

### Immunohistochemistry

Immunohistochemical analysis was performed according to standard protocols (see below), on a series of 14 μm-thick cryosections. Antibodies against Cleaved-Caspase-3, apoptosis-inducing factor (AIF), Cyclin-dependent-kinase 4 (CDK4), BCL2 Associated X, Apoptosis regulator (BAX), Receptor Interacting Serine/Threonine Kinase 3 (RIPK3) and the H3K27me3 antigens were used (Table 1). Slides were incubated 1h with the blocking solution containing 10% Normal Goat Serum (NGS) and 0.3% TritonX-100, followed by overnight incubation with the primary antibody diluted in blocking solution, and then washed 3 times with PBS. Appropriate fluorescent secondary antibody was incubated for 1h at room temperature (Table 1). Nuclei were counter-stained with 4,6-diamidino-2-phenylindole (DAPI). When comparison of fluorescence was made between groups (for instance in Figure 3), pictures were taken with the same acquisition time for a specific labeling (in this figure, H3K27me3: 900 ms, AIF, 700 ms and DAPI: 25 ms).

**TABLE 1:** List of primary and secondary antibodies for immunochemistry.

	Species	Source	Working dilution/incubation time
**Primary antibody**			
M/L Opsin	Rabbit	Chemicon	1/2000 ON
Cdk4	Rabbit	Santa Cruz, SC-601	1/100 ON
H3K27me3	Rabbit	Merck Millipore #07-449	1/200 ON
Cleaved Caspase-3	Rabbit	Cell Signaling Technology #9661	1/250 ON
AIF	Rabbit	Abcam #ab32516	1/250 ON
BAX	Rabbit	Cell Signaling	1/200 ON
RIPK3	Mouse	Labforce	1/50 ON
**Secondary antibody**			
Alexa Fluor 633 Goat anti-Rabbit IgG Biotinylated anti-rabbit IgG (H+L)	**Goat Goat**	Invitrogen A21072 Vector BA1000	1/2000, 1 h, RT 1/200

*ON = overnight, RT = room temperature.*

#### H3K27me3 and AIF Labelings

An antigen retrieval treatment was performed prior to antibody incubation. The cryosections, in a 10 mM citrate buffer at pH 6.0, were first heated in a microwave oven 5 min at 696 W and then cooled down for 30 min at room temperature (RT).

H3K27me3 fluorescence intensity (Supplementary Figure S2) was quantified using Fiji (ImageJ). The images were cropped to select only the ONL, then the same intensity threshold was applied to all the pictures, and the mean intensity was automatically measured in the area above threshold. Some nuclei in the RD proximity picture were saturated, leading to an underestimation of the mean intensity on this picture.

#### CDK4 Labeling

To amplify CDK4 signal, a DAB staining was performed with a previous anti-peroxidase agent (0.3% H_2_O_2_ for 5 min at RT). After primary antibody incubation, biotinylated goat anti-rabbit antibody was added for 1h and then the ABC substrate for 30 min. DAB revelation lasted 2 min at RT.

#### Active Caspase-3 Staining

The Cleaved-Caspase-3 staining (Cell Signaling Technology #9661, Table 1) was performed according to standard protocol, with one hour blocking with 5% NGS.

#### TUNEL Staining

To visualize dying cells on sections, a TUNEL assay kit conjugated with TMR (Terminal deoxynucleotidyl Transferase Biotin-dUTP Nick End Labeling) was performed following instructions (Roche Diagnostics, Basel, Switzerland). Negative controls consisted of omitting the terminal deoxynucleotidyl transferase enzyme from the labeling, and gave no staining at all. Cells with a homogenous nucleus staining the size of the whole nucleus, or a little shrunk, or with a circle staining (corresponding probably to the euchromatin), were taken into account as previously described in Sahaboglu et al., 2013.

### Statistical Analysis

Positive cells were counted in the most central retina sections. All measurements were taken by Olympus BX6 fluorescein microscopy. The quantification of positive cells was performed on the pictures from 10 different slices for each sample. Because after 3 days *in vitro*, the shape of the retinal tissue can change, we always considered (1) areas which were well layered and where no confusion could be made between ONL and INL cells, and (2) homogeneous areas in a full 40x field. All the data are expressed as mean plus or minus the standard error of the mean (SEM). Data were analyzed using the Mann-Whitney *U*-test and the *p*-value given.

## Results

### Number of Photoreceptor Layers

In all explants, the number of photoreceptor layers was quite stable in both groups and at different time points (Table 2 and Figure 2C). Some heterogeneity exists within the same tissue sample, but each group contained retina with similar numbers of photoreceptor rows (Figure 2). We checked the presence of cones and found M/L-OPSIN-positive cells from 1 to 7 days of the culture period (data not shown). With time, many cells had M/L-OPSIN mislocalization suggesting a degenerative process. However, we did not investigate the rate of the cone degeneration because of the heterogeneity of the samples received, which were often from the retinal periphery region.

**TABLE 2:** Temporal expression of cell death markers during the culture of human retina explants with and without RPE.

	Treatment	1 Div		3 Div		5 Div		7 Div	
					
		Average *n* = 4	*p*	Average *n* = 4	*p*	Average *n* = 4	*p*	Average *n* = 4	*p*
PR rows	−RPE	8.3	*0.76*	7.8	*0.88*	7.0	*0.61*	8.1	*0.77*
	+RPE	7.5		7.2		6.4		7.7	
CDK4	−RPE	1.95	*0.02*	3.70	*0.03*	3.36	*0.1*	4.70	*0.014*
	+RPE	0.00		1.62		1.70		1.75	
H3K27me3	−RPE	1.73	*0.24*	2.67	*0.61*	11.95	*0.014*	10.09	*0.014*
	+RPE	1.63		2.72		2.92		3.93	
TUNEL ONL	−RPE	2.63	*0.04*	11.83	*0.014*	11.03	*0.04*	16.30	*0.014*
	+RPE	1.16		1.36		4.36		7.98	
TUNEL INL*	−RPE +RPE	3.9 4.1	/	9.6 6.5	/	11.7 4.3^#^	/	8.7 3.3	/
AIF*	−RPE	0.7	/	5.7	/	0.3	/	0	/
	+RPE	0.2		0.6		0		0	

* : *n* = 2, ^#^: *n* = 1, PR = photoreceptors. Note the transient expression of AIF as previously documented and the delayed appearance of the H3K27me3 mark. The peak of TUNEL-positive cells in the ONL rises at 3 DIV and remains elevated until 7 DIV at least.

### TUNEL Staining; Cell Death Marker

**FIGURE 1:**
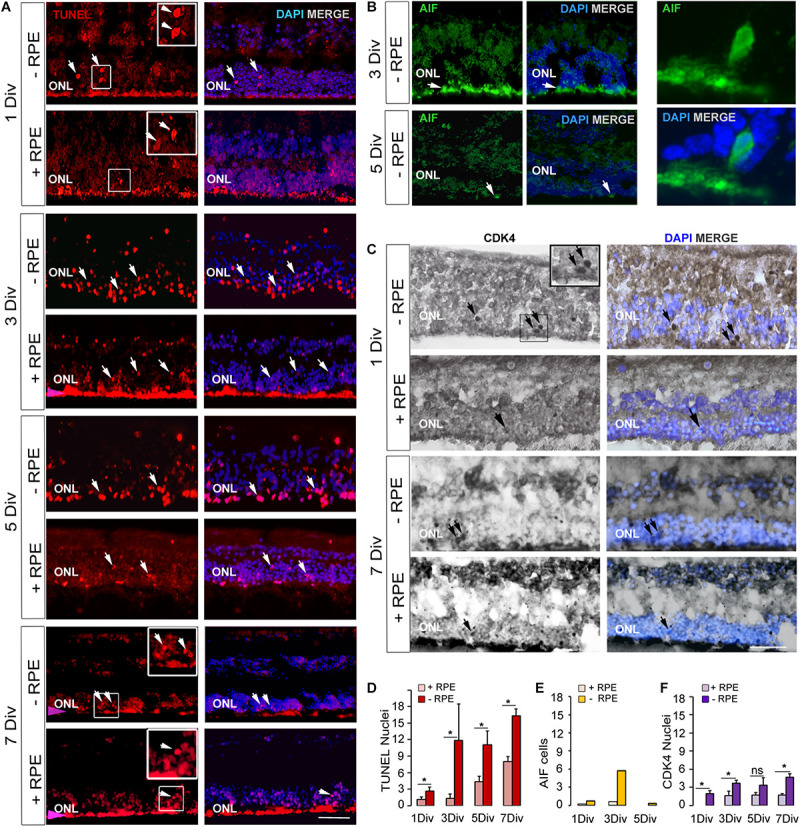
Expression of cell death markers on human retinal explants, an *in vitro* model of retinal detachment. **(A,D)** TUNEL-positive cells imaging (arrows) and counting at 1 DIV, 3 DIV, 5 DIV, 7 DIV in RD group (-RPE) and control group (+RPE). The presence of RPE produced an artifact red fluorescent background (pink arrow). **(B,E)** Imaging and counting of AIF-positive cells, which are present at 3 DIV in RD group (-RPE), but are rare at 5 DIV (arrow). A representative AIF-positive cell is shown at high magnification. **(C,F)** CDK4 positive cells imaging (arrows) and counting in RD group (-RPE) and control group (+RPE), at 1 DIV and 7 DIV. DIV: Days *in vitro*, ONL: outer nuclear layer, RPE: retinal pigment epithelium. Calibration bar: 20 μm.

TUNEL-positive cells were present from the beginning of the experiment at a low level in the control group despite the different postmortem delays (12, 14, 23, and 24 h) and the dissection procedure of the tissue. The number of TUNEL-positive cells significantly and markedly increased after 3 days *in vitro* (DIV) and remained elevated until 7 DIV. An increase of more than 10-fold of TUNEL-positive cells was observed at 3 DIV between the RD and the control groups (*p* = 0.014) (Table 2 and Figures 1A,D). Interestingly, this augmentation of cell death coincides with what has been described for rhegmatogenous RD *in vivo* (Hisatomi et al., 2001; Arroyo et al., 2005). A significant 38% increase of TUNEL-positive cells continued to occur between 3 DIV and 7 DIV (Table 2 and Figures 1A,D). We then investigated the presence of potential actors of the cell death process. Note that the TUNEL-positive cells also appeared in the INL (Figure 1A and Table 2), and their number is increased at all time points, by the absence of the RPE.

**FIGURE 2:**
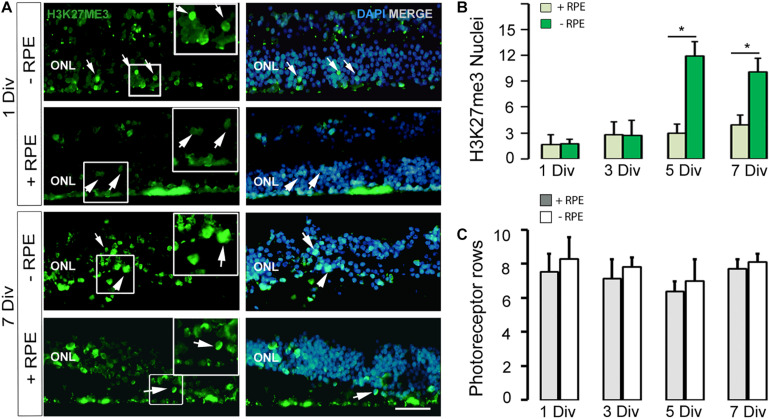
**(A,B)** Imaging and counting of H3K27me3-positive cells at 1 DIV and 7 DIV, in the human *in vitro* model of RD (-RPE) and control (+RPE). Note the marked increased number of H3K27me3-positive cells at 5 DIV **(B)** and 7 DIV in the RD group (-RPE). **(C)** The number of photoreceptor rows is stable all along the culture system in the two groups (RD model +RPE and control -RPE). DIV: Days *in vitro*, ONL: outer nuclear layer, RPE: retinal pigment epithelium. Calibration bar: 20 μm.

**FIGURE 3:**
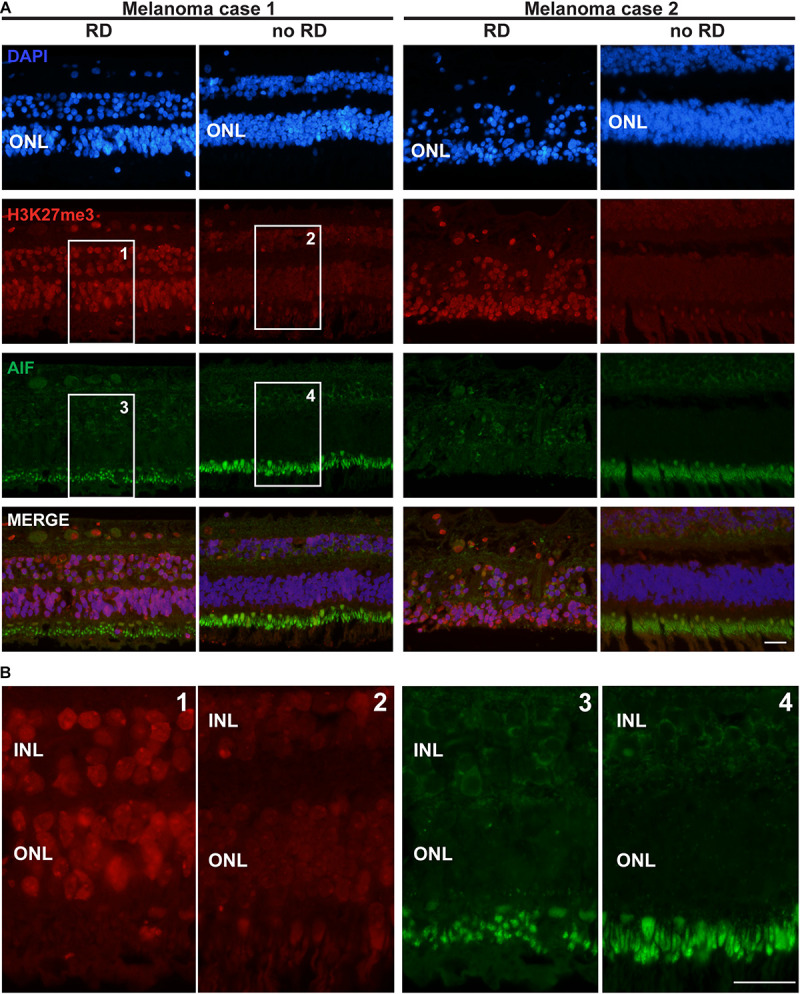
H3K27me3 mark distribution in a retina of two patients with retinal detachment (RD) due to tumor growth. **(A)** The first row of pictures showing the retina nuclei evidenced by DAPI staining indicates that in the retinal detachment (RD) area, the cell density is weaker in the ONL and this layer is thinner for patient 2 compared to the control area. The H3K27me3 staining shows a markedly stronger level in the RD area (see also magnification in Panel **B**) in comparison to the intact area. AIF labeling is absent in all ONL samples, but highly present in the inner segments of rods and cones (better shown in panel **B**). A light expression is also present around some nuclei of INL cells. The fourth row presents the merged labeling of the three previous stainings. **(B)** High magnification of the insets presented for H3K27me3 and AIF labeling in panel **(A)**. Note the altered size of the inner segments in the detachment area as suggested by the AIF labeling (probably present in the mitochondria of this organelle). RD: Retinal detachment, ONL: outer nuclear layer, INL: inner nuclear layer. Calibration bar: 25 μm.

### Neither the Canonical Apoptotic Pathway, nor Necrosis Is Activated in the Human RD Group

Cleaved-Caspase-3 is a final effector of apoptosis (Slee et al., 2001; Kim et al., 2002; Zacks et al., 2003; Murakami et al., 2013) and was chosen to reveal the extent of apoptotic events in the retina with and without RPE. Surprisingly, very rare positive cells in the ONL were present in both groups and no peaks of apoptosis were identified (data not shown). In the INL, an average of 0.6 positive cell per 40× field was observed in explants without RPE at 3 DIV (*n* = 1), none at 5 DIV (*n* = 1), and 0.3 at 7 DIV (*n* = 2). BAX was not found in the ONL of any explant at any time point. A few BAX-positive cells were observed in the INL: none at 1 DIV in both groups and an average of 0.9 positive cell per field was present at 7 DIV in explants with RPE, and none in explants without RPE (*n* = 3). RIPK3, a marker of necrosis, was also absent from the ONL of both groups. Only some cells in the retinal ganglion layer were observed after 7 days *in vitro* without RPE (Supplementary Figure S1). RIPK3 was also absent from the INL of explants with and without RPE at 1 and 7 DIV (*n* = 1). However, the technique sensitivity may have revealed only cells with highly expressed antigens and thus may underestimate the total number of positive cells.

### Apoptosis-Inducing Factor (AIF) Staining

Apoptosis-Inducing Factor is normally localized in mitochondria membranes. During cell death, AIF is released and translocated to the nucleus (Susin et al., 1999; Hisatomi et al., 2001, 2008). AIF can be released from mitochondria either in a caspase-dependent or caspase-independent way (Sanges and Marigo, 2006) and can mediate caspase-independent cell death (Sanges and Marigo, 2006). We observed that AIF was present in cell nuclei in the ONL at 1 DIV and with a marked peak at 3 DIV in the RD group. AIF-positive cells augmented when TUNEL-positive cells increased at 3 DIV (Table 2 and Figures 1B,E). The increase was about 10-fold in comparison to the control group (*n* = 2). At 5 DIV and 7 DIV there were almost no visible AIF-positive cells. No AIF-positive cells were detected in the INL at any time point in explants with RPE (*n* = 1) or without RPE (*n* = 3).

### The Cell Cycle Marker CDK4 Is Moderately Expressed During the Degenerative Process in Absence of RPE

No CDK4 was observed in the photoreceptor nuclei of the control group after 1 DIV and only very few cells expressed this protein between 3 and 7 DIV. In the RD group, CDK4-positive cells were present starting at 1 DIV (Table 2 and Figures 1C,F); their number moderately increased afterwards and remained stable between 3 and 7 DIV. Comparison between the two groups showed statistical difference at each time-point, except for 5 DIV (Table 2 and Figure 1F). Note that the numbers of CDK4-positive cells are much lower in comparison to TUNEL-positive ones (Table 2).

### Epigenetic Modifications of the H3K27me3 Mark Occur at the Late Stage of the Degenerative Process

Recent works described epigenetic modifications during the course of retinal degeneration (Wahlin et al., 2013; Corso-Díaz et al., 2018) with a small increase of the H3K27me3 mark level in retina extract (Zheng et al., 2018). As we also observed that epigenetic modifications occur at the histone level in some rodent models of retinitis pigmentosa (unpublished data), we investigated the H3K27me3 mark known to regulate gene repression (Wahlin et al., 2013). In the control group, the number of H3K27me3-positive cells in the ONL remained low until 5 DIV and showed a slight elevation at 7 DIV, whereas an important increase of this mark was observed at 5 DIV in the RD group and stayed elevated at 7 DIV, although partially reduced (Table 2 and Figures 2A,B). Note that the elevated number of H3K27me3-positive cells appears after the peak of TUNEL-positive cells at 3 DIV.

### The H3K27me3 Mark Is Present in Photoreceptors of Detached Retina

Seizing the opportunity to obtain sections from 4 eye globes containing a uveal melanoma resulting in retinal detachment, we investigated the distribution of the H3K27me3 mark in these retinas. In the attached region close to the center of the retina, the H3K27me3 was present in photoreceptors and homogenously distributed. In the area where the detachment was the most noticeable (mid-periphery, at the opposite side of the optic nerve) and where the retina was thinner, many photoreceptors expressed high level of H3K27me3. To ensure that such difference in the mark presence was not due to retinal area or thickness, we also looked at the periphery where the retina was of the same size and not detached by the tumor. We observed a discrete H3K27me3 labeling as described for the non-detached central part. The Figure 3 presents two representative cases out of four with the same pattern. The intensity of the H3K27me3 fluorescence signal was quantified in the ONL shown in the Supplementary Figure S2 and provides the following results: no RD fluorescence mean intensity = 107.8; RD mean intensity = 146.1 and no RD periphery mean intensity = 108.5. Interestingly, more TUNEL-positive cells were observed in the thin retina area close to the tumor, where the H3K27me3 level was high (Supplementary Figure S3). Two samples showed several dying cells in the ONL and, remarkably, the upper INL also contained TUNEL-positive cells (5 and 8 TUNEL-positive cells per slice in INL in each positive sample in the detached area).

No AIF labeling in the nucleus of ONL cells was detected (Figure 3). Unfortunately, we were no longer able to check the expression of CDK4 in these samples, due to human-CDK4 antibody issues (Santa Cruz antibody production discontinued). However, we used previous material from monkeys (*Macaca fascicularis* eyes). We performed subretinal injections with TSSM (vehicle) as a control group for a gene therapy study and to complete the present work we evaluated whether the retina detachment, which led to some very local tissue thinning, was correlated to CDK4 expression and the presence of the H3K27me3 mark. The CDK4 labeling was made previously and we added labeling for H3K27me3. The detached area displayed a higher level of H3K27me3 marks after 90 days in comparison to the non-detached area. Some very rare CDK4-positive cells were observed (Supplementary Figure S4). The ONL size cannot be compared, because the samples are not from the same regions.

## Discussion

The present data open new perspectives to test *in vitro* different drugs to block a specific cell death pathway, at several time points of the degenerative process, in order to optimize cell survival. This work reveals the presence of various potential actors of cell death and the comprehension of their mechanisms is still necessary to correctly propose a protective approach for the injured photoreceptors.

A clear demonstration of apoptosis as a major form of photoreceptor cell death after RD was made by Cook and colleagues in a cat model of RD (Gavrieli et al., 1992; Cook et al., 1995). They showed that photoreceptor cells in the detached retina exhibit strong TUNEL reactivity as well as pyknotic morphological changes. Moreover, they demonstrated that the photoreceptor cell death after RD occurs within an earlier period than previously recognized: TUNEL-positive cells are detected as early as 1–3 days after RD, followed by a decline in their number over the next few weeks. This early activation of cell death, has been confirmed in other animal models of RD with rhegmatogenous RD (Hisatomi et al., 2001; Arroyo et al., 2005).

In our *in vitro* model of human RD, we noticed the peak of TUNEL-positive cells at 3 days *in vitro* (DIV), with a plateau until 7 DIV (latest time-point), thus underlining the need for urgent surgery in patients affected by retinal detachment. Surprisingly, a post-mortem delay inferior to 24 h (12, 14, 23, and 24 h) had little effect on photoreceptor death, probably because a minimal metabolism is maintained between the RPE and the photoreceptors. We also noticed cell death in the INL, mostly in the absence of the RPE. A similar observation, but with fewer TUNEL-positive cells, was made in two eye globes (out of four) where the retinal detachment occurred after tumor growth. The tumor may have compromised the retina vessel blood flow in those cases. These data suggest that the culture conditions recapitulate some features of the *in vivo* retinal detachment, but the environment does not contain immune cells and factors that may be secreted after prolonged retina detachment. In addition, the lack of blood flow may also influence the rate of photoreceptor death.

Several works revealed in animal models that Caspase-3 is activated during some forms of retinal degeneration (Wenzel et al., 2005; Sancho-Pelluz et al., 2008; Trichonas et al., 2010; Murakami et al., 2013). We thus investigated the course of Cleaved-Caspase-3 activation in our *in vitro* model. Surprisingly in both groups, only few cells were positive for this enzyme suggesting alternative pathways conducting the sensory cells to death.

However, the AIF expression pattern resembles that described after retinal detachment in rodents as well as in human samples (Hisatomi et al., 2008). Interestingly, the peak of AIF parallels the first elevated raise of dying photoreceptors suggesting a role of AIF in this early degenerative process. The appearance of AIF is only transitory whereas the cell death process is still continuing, implying that other actors are involved in this process. Nonetheless, these results suggest that the first wave of degenerating photoreceptors requires appropriate and specific drugs to block AIF action in order to enhance the survival of the retina. They also support previous studies in rodents showing that inhibitors of the HIV protease, nelfinavir and ritonavir, can act as anti-cell death factors, impeding, in the photoreceptors, the translocation of AIF into the nucleus, and suggest that such mechanisms may also be involved in the RD in human retina (Hisatomi et al., 2008).

We also investigated whether a factor involved in the necrosis process, RIPK3, was expressed during the retinal degeneration phase. No positive cells were observed in the ONL, but some appeared in the GCL at a late stage of the culture without RPE, suggesting that retinal detachment does not stimulate necrosis in photoreceptors. A similar observation was made in the retinas detached due to melanoma tumors.

Concerning the potential factors that may be responsible for the continuation of cell death, we investigated whether proteins of the cell cycle were re-expressed as observed in several models of retinal degeneration (Vincent et al., 1996; Yang et al., 2001; Nguyen et al., 2003; Höglinger et al., 2007). We previously identified that CDK4 is reactivated in various rodent models of inherited retinal dystrophies as well as during light damage (Zencak et al., 2013) and plays a role in the execution phase of photoreceptor death (Zencak et al., 2013). In control explants, a low number of CDK4-positive photoreceptors was present in all time points examined starting at 3 DIV. In the RD group, CDK4 was significantly more expressed in all time points, but the kinetics of CDK4 appearance did not correlate or precede the peak of cell death observed at 3 DIV. Moreover, the number of CDK4-positive cells was much lower than the number of TUNEL-positive cells. This observation differs from those obtained in rodent models of retinal dystrophies (Zencak et al., 2013). These results suggest that CDK4 is probably not involved in the first peak of cell death at 3 DIV and may little account for the continuation of photoreceptor degeneration at later time points. In this situation, we cannot exclude that some microglia may also express CDK4.

We found that the H3K27me3 mark showed a massive increase at 5 DIV in the RD group, in contrast with the control group where the level was very low. Epigenetic modifications have been poorly documented so far in retinal diseases, nonetheless interesting changes in DNA methylation were observed in retinas of different mouse models of retinal degeneration. DNA methylation is generally associated with repression of transcription (Jones and Liang, 2009) and was recently observed to be increased in dying photoreceptors (Farinelli et al., 2014). The TUNEL assay for dying cells co-labeled the 5mC-positive cells (anti-5-methylcytosine antibody was used to analyze photoreceptor DNA methylation *in situ*) to a great extent, suggesting an intimate connection between increased DNA methylation and the degeneration of the photoreceptors (Chang et al., 2002; Kaur et al., 2011; Sahaboglu et al., 2013; Farinelli et al., 2014). DNA methylation was found to be most prominent during the final stages of cell death (Farinelli et al., 2014). However, it cannot be excluded that hypermethylation of specific genes may be starting far earlier (Farinelli et al., 2014). Our positive results for the H3K27me3 mark well correlate with DNA methylation in photoreceptors. Indeed, EZH2, the enzyme that methylates H3 on lysine 27, recruits the DNA-methyl transferase (DNMT) necessary for DNA methylation (Viré et al., 2006) suggesting that EZH2 first initiates epigenetic changes which are then reinforced by DNMTs. EZH2 belongs to the Polycomb repressive complex-2 (PRC2) and interestingly, BMI1, a protein of PRC1 often acting after PRC2, was identified to be crucial during photoreceptor death (Zencak et al., 2013). H3K27me3 staining may thus be more relevant to identify genes involved in the process of cell death.

However, in the present study, the highest peak of H3K27me3-positive cells is expressed at 5 DIV, suggesting that the first wave of photoreceptor death is independent of EZH2 activity and that the continuation of the degeneration may be related to EZH2. Supporting this hypothesis the anti-apoptotic agent Z-VAD reduced the number of TUNEL-positive cells in a rat model of RD, but not photoreceptor loss (Murakami et al., 2013), suggesting other death mechanisms. The histological analysis of retinal detachment due to tumor growth showing high level of H3K27me3 supports also a potential role for EZH2 in photoreceptor death, in pathophysiological conditions *in situ*. Interestingly, a higher expression of H3K27me3 was also found in the photoreceptor layer of a monkey retina, 90 days after detachment, suggesting that this injury induced a long term change of the cell homeostasis, the consequence being enigmatic at this stage although rare CDK4-positive cells were observed. This *in vitro* work needs to be confirmed by future studies with specific inhibitors to prevent photoreceptor death at different injury stages. This should reveal whether the medication to slow down photoreceptor degeneration has to be adapted to each stage of the degenerative process. Such studies were not possible yet due to the low number of samples obtained each year.

These data also support the clinical observations highlighting the importance to take in charge the patient to perform RD surgery within the first 3 days, in order to optimize for the best functional recovery. In view of our and other previously published results, successive death pathways are activated after retinal detachment, necessitating adapted therapies for each disease stage to protect the retina.

## Conclusion

This study demonstrates the relevance of human retina explant cultures to model retinal detachment. Furthermore our results suggest that the cell death due to retinal detachment is controlled by different features including AIF during the early photoreceptor death wave, whereas epigenetic modifications, controlled by the H3K27me3 mark, may account for the late phase of the degenerative process. Further studies need to identify which gene expressions are affected by such modifications and if drugs inhibiting these pathways may have a protective action on photoreceptors during RD.

## Data Availability Statement

The raw data supporting the conclusion of this article will be made available by the authors, without undue reservation.

## Ethics Statement

The studies involving human participants were reviewed and approved by Commission cantonale d’éthique de la recherche sur l’être humain (CER-VD) protocol No 340-15. Written informed consent for participation was not required for this study in accordance with the national legislation and the institutional requirements.

## Author Contributions

YA designed the experiments, performed the analyses and wrote the manuscript. MM, DW, and AM participated to the experiments and analyses. JP performed the experiments and wrote the manuscript. AB performed experiments and the analyses and contributed to the manuscript writting. CK and AM performed experiments and critically revised the manuscript. FB-C performed experiments and managed the human tissue access and the biobank and wrote the manuscript. All authors contributed to the article and approved the submitted version.

## Conflict of Interest

The authors declare that part of the research was included in a patent (WO2020/011607A1) and could be construed as a potential conflict of interest. The authors declare that the research was conducted in the absence of any commercial or financial relationships that could be construed as a potential conflict of interest.

## Supplementary Material

Supplementary Figure 1 BAX and RIPK3 expression in human retina explants with and without RPE. BAX expression was very rarely observed in both groups. An example of BAX labeling is presented for the 1 DIV time point **(upper left panel)**. RIPK3 expression was observed in the retinal ganglion cell layer of retina cultivated without RPE at 7 DIV without RPE **(lower right panel)**. DIV: Days *in vitro*, ONL: outer nuclear layer. Calibration bar: 25 μm.

Supplementary Figure 2 H3K27me3 mark labeling in a retina of one patient (case 3) with retinal detachment (RD) due to tumor growth, whose intensity has been quantified (see body text). The left and right columns show the homogeneous presence of the H3K27me3 mark in all retinal layers. The central column shows a markedly higher expression of the H3K27me3 mark in the ONL in the RD area. Note that also some cells in the INL are well positive. The DAPI staining also appears brighter, probably due to chromatin condensation induced by the H3K27me3 mark. RD: Retinal detachment, ONL: outer nuclear layer, INL: inner nuclear layer, GCL: ganglion cell layer. Calibration bar: 25 μm.

Supplementary Figure 3 TUNEL-positive cells are present in the detached retina area of the patient case 1. TUNEL-positive cells (red) were detected in the detached retina area above the melanoma (upper right picture) and not in the attached region (upper left picture). The corresponding nucleus stainings (DAPI, blue) are presented below. The merged pictures reveal that the great majority of the TUNEL-positive cells are present in the ONL. The stars show two red spots in the INL corresponding to two red blood cells (autofluorescence and no DAPI staining). RD: Retinal detachment, ONL: outer nuclear layer. Calibration bar: 25 μm.

Supplementary Figure 4 H3K27me3 mark is present in ONL cells long time after retinal detachment. No detection of the H3K27me3 mark is visible in the non-detached retina, whereas 90 days after RD, the detached retina presents punctuated labeling of the mark. The second and third rows of pictures present the retina nucleus (DAPI staining) and the merged pictures. Note the reduced number of photoreceptor in the RD group. The last panels show a regular expression of CDK4 in the INL (probably in the Muller cells nuclei), whereas the ONL is devoid of its expression. Rare CDK4-positive cells were detected in the ONL of detached retina. RD: Retinal detachment, ONL: outer nuclear layer. Calibration bar: 50 μm.
