# Supplementary figures and images for: An in vitro Model of Human Retinal Detachment Reveals Successive Death Pathway Activations
Source: Front Neurosci. 2020 Nov 26;14:571293. doi: 10.3389/fnins.2020.571293 (PMC7726250; doi:10.3389/fnins.2020.571293)

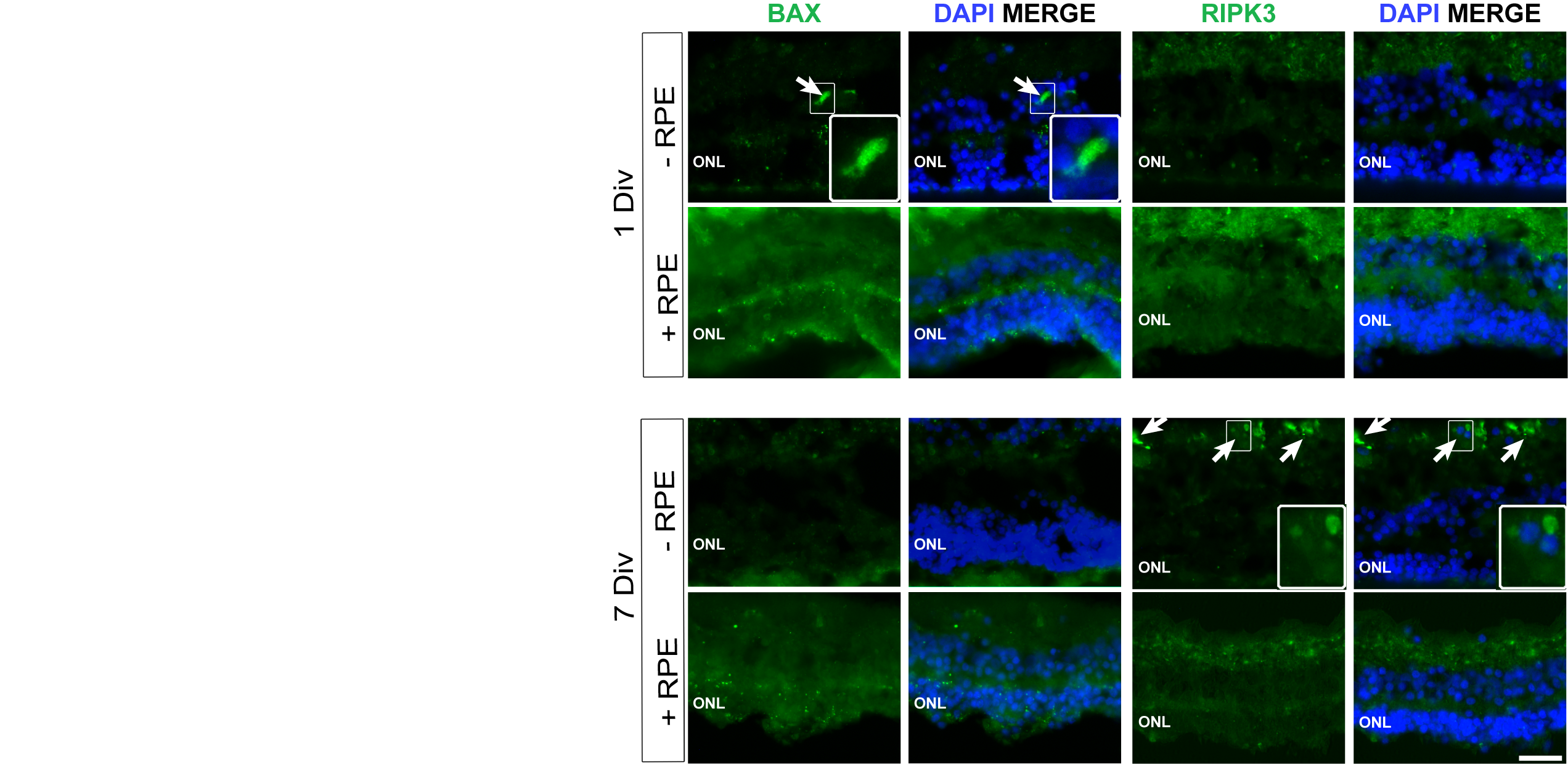

Supplement: Supplementary Figure 1 — BAX and RIPK3 expression in human retina explants with and without RPE. BAX expression was very rarely observed in both groups. An example of BAX labeling is presented for the 1 DIV time point (upper left panel). RIPK3 expression was observed in the retinal ganglion cell layer of retina cultivated without RPE at 7 DIV without RPE (lower right panel). DIV: Days in vitro, ONL: outer nuclear layer. Calibration bar: 25 μm. [file Image_1.tif]

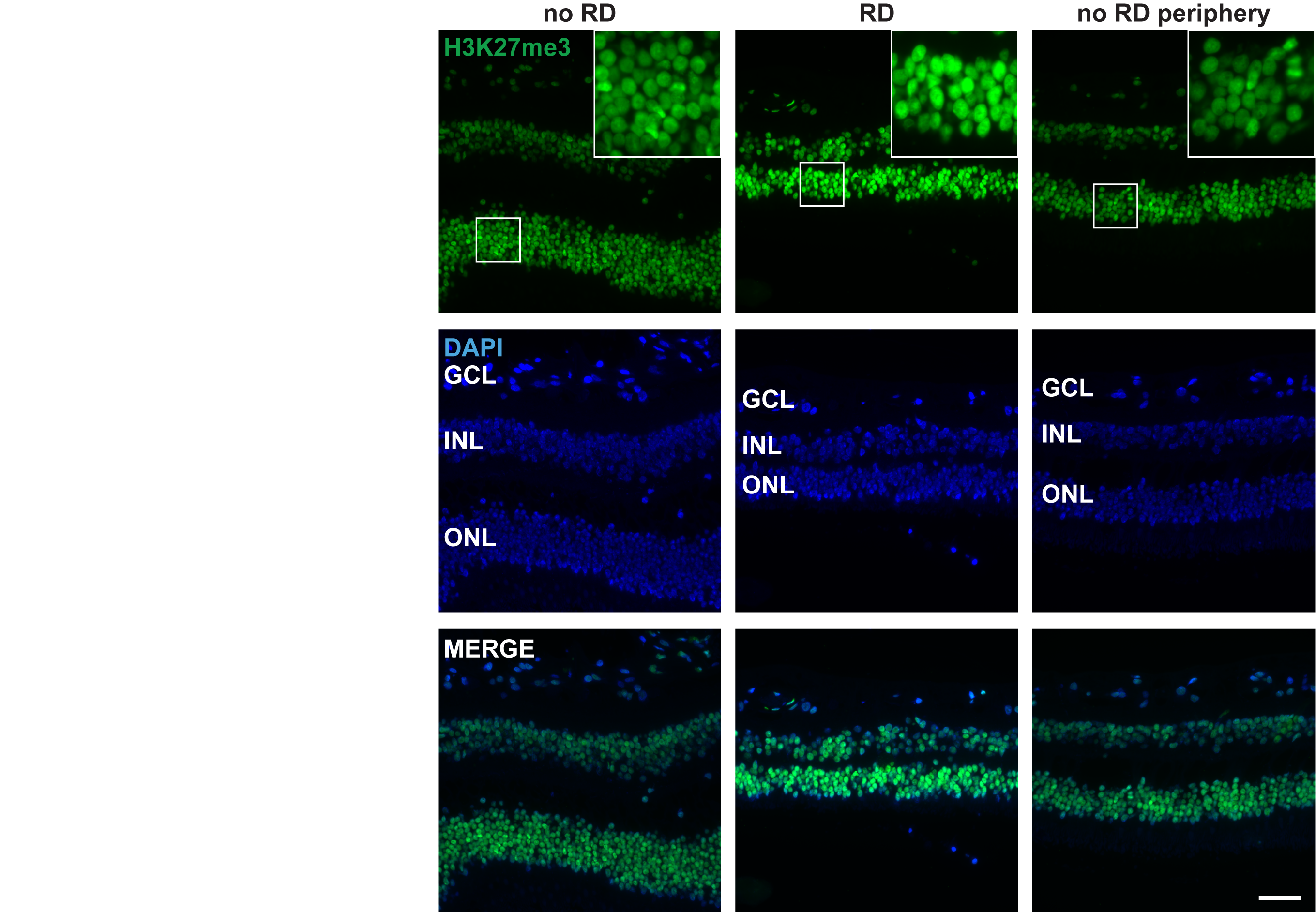

Supplement: Supplementary Figure 2 — H3K27me3 mark labeling in a retina of one patient (case 3) with retinal detachment (RD) due to tumor growth, whose intensity has been quantified (see body text). The left and right columns show the homogeneous presence of the H3K27me3 mark in all retinal layers. The central column shows a markedly higher expression of the H3K27me3 mark in the ONL in the RD area. Note that also some cells in the INL are well positive. The DAPI staining also appears brighter, probably due to chromatin condensation induced by the H3K27me3 mark. RD: Retinal detachment, ONL: outer nuclear layer, INL: inner nuclear layer, GCL: ganglion cell layer. Calibration bar: 25 μm. [file Image_2.tif]

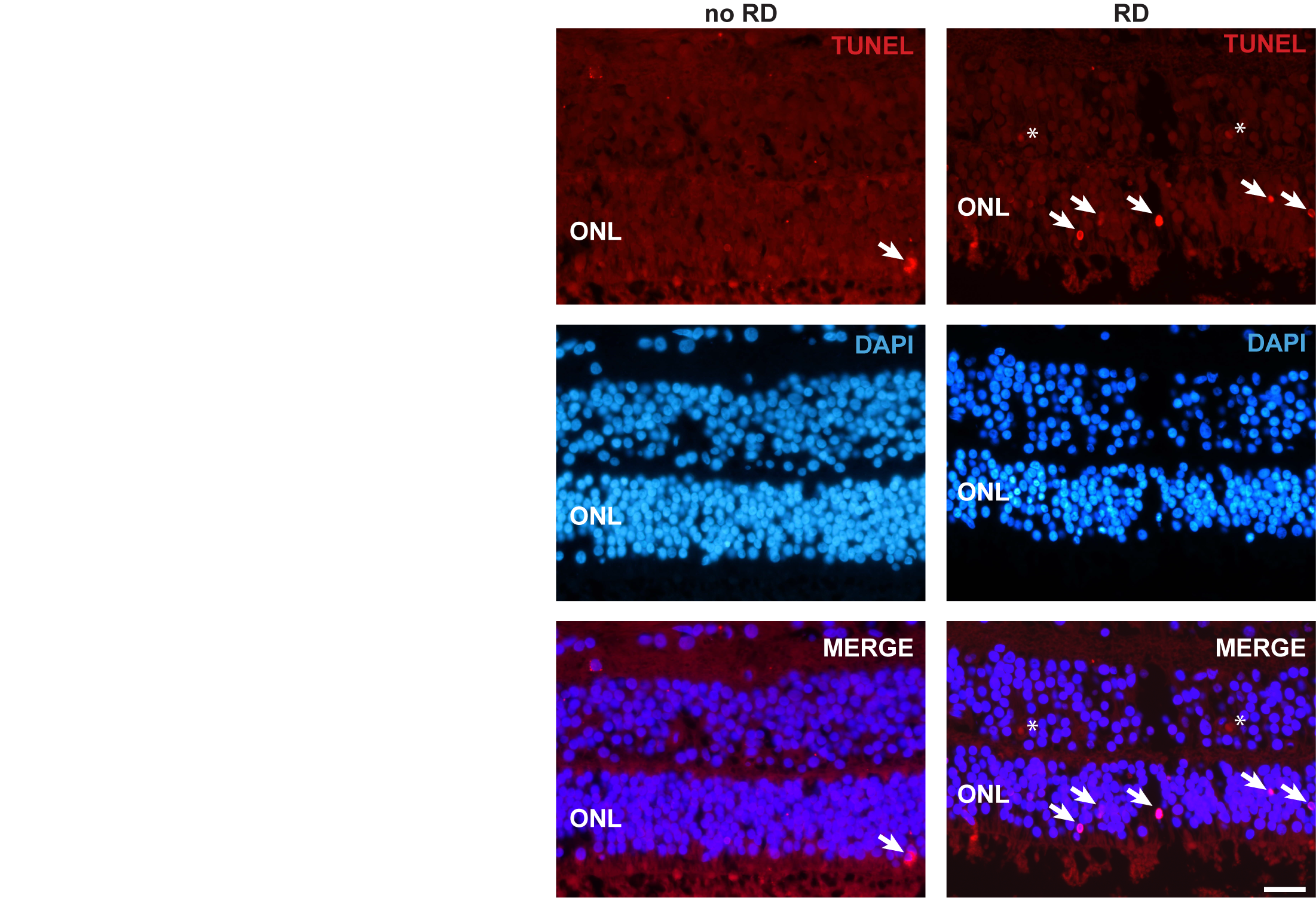

Supplement: Supplementary Figure 3 — TUNEL-positive cells are present in the detached retina area of the patient case 1. TUNEL-positive cells (red) were detected in the detached retina area above the melanoma (upper right picture) and not in the attached region (upper left picture). The corresponding nucleus stainings (DAPI, blue) are presented below. The merged pictures reveal that the great majority of the TUNEL-positive cells are present in the ONL. The stars show two red spots in the INL corresponding to two red blood cells (autofluorescence and no DAPI staining). RD: Retinal detachment, ONL: outer nuclear layer. Calibration bar: 25 μm. [file Image_3.tif]

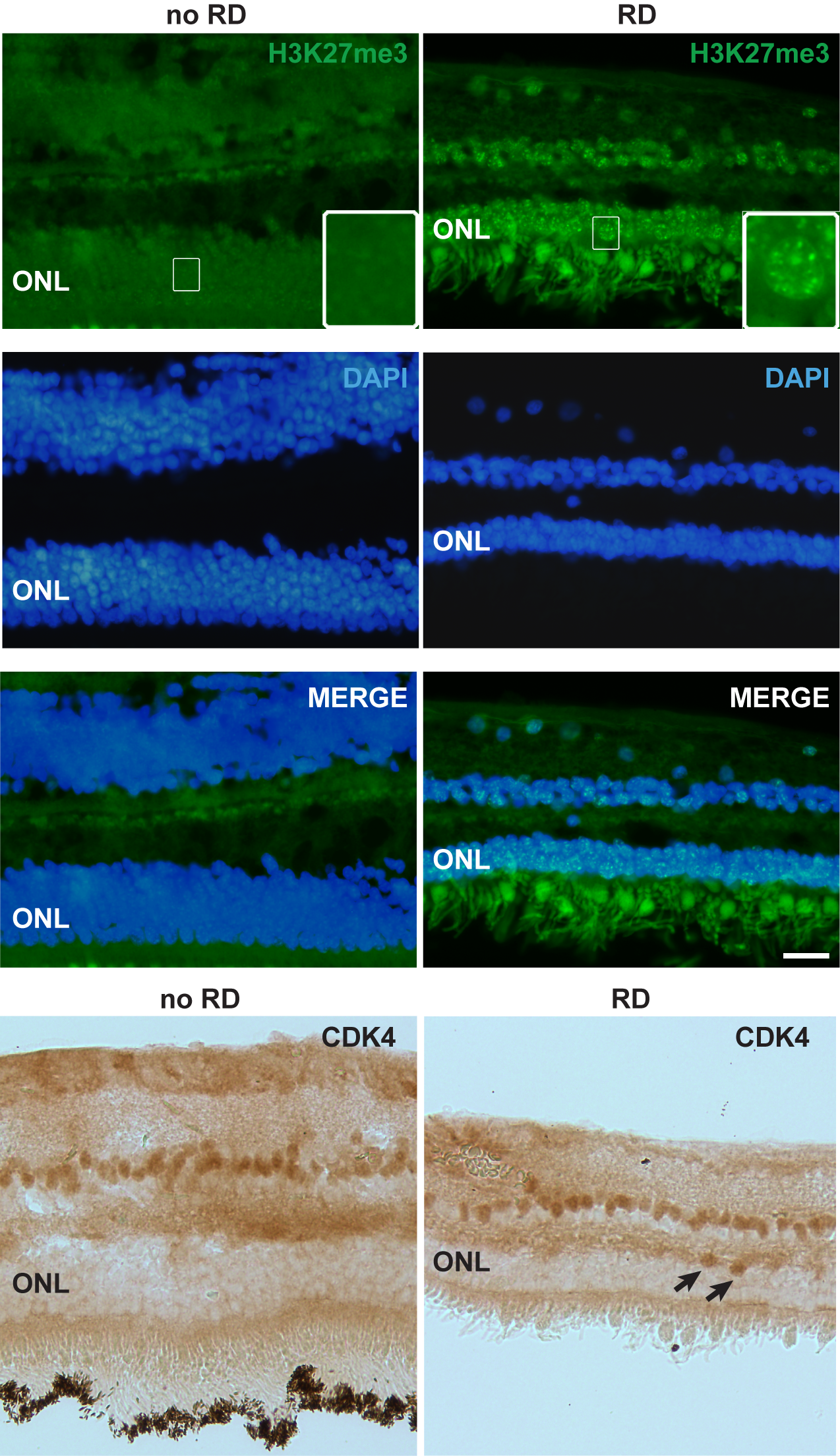

Supplement: Supplementary Figure 4 — H3K27me3 mark is present in ONL cells long time after retinal detachment. No detection of the H3K27me3 mark is visible in the non-detached retina, whereas 90 days after RD, the detached retina presents punctuated labeling of the mark. The second and third rows of pictures present the retina nucleus (DAPI staining) and the merged pictures. Note the reduced number of photoreceptor in the RD group. The last panels show a regular expression of CDK4 in the INL (probably in the Muller cells nuclei), whereas the ONL is devoid of its expression. Rare CDK4-positive cells were detected in the ONL of detached retina. RD: Retinal detachment, ONL: outer nuclear layer. Calibration bar: 50 μm. [file Image_4.tif]
